# Early rapid weight gain and subsequent overweight and obesity in middle childhood in Peru

**DOI:** 10.1186/s40608-016-0135-z

**Published:** 2016-12-13

**Authors:** Mary E. Penny, M. Michelle Jimenez, R. Margot Marin

**Affiliations:** Instituto de Investigación Nutricional, Ave La Molina 1885, La Molina, Lima 12 Peru

**Keywords:** Early rapid weight gain, Infancy, Overweight and obesity, Peru, Childhood

## Abstract

**Background:**

Rapid postnatal weight gain is associated with risk of overweight and obesity, but it’s unclear whether this holds in populations exposed to concurrent obesogenic risk factors and for children who have been extensively breastfed. This study investigates whether an increase in weight for age from birth to 1 year (infancy) and from 1 to 5 years (early childhood) predicts overweight and obesity, and waist circumference at 8 years, using data from a longitudinal cohort study in Peru.

**Methods:**

Generalized estimating equations (GEE) models were constructed for overweight and obesity, obesity alone and waist circumference at 8 years versus rapid weight gain in infancy, and early childhood including adjusted models to account for confounders.

**Results:**

Rapid weight gain in both periods was associated with double the risk of overweight and obesity, obesity alone at 8 years and increased waist circumference even after controlling for maternal BMI and education level, sex of child, height-for-age at 8 years, consumption of “fast food” and number of days of active exercise. The association was significant, with some differences, for children in both rural and urban environments.

**Conclusions:**

Rapid weight gain in infancy and in early childhood in Peru is associated with overweight and obesity at age 8 years even when considering other determinants of childhood obesity.

**Electronic supplementary material:**

The online version of this article (doi:10.1186/s40608-016-0135-z) contains supplementary material, which is available to authorized users.

## Background

Childhood overweight and obesity, which are strongly associated with adult overweight and increased risk of non-communicable disease [1], are prevalent and increasing in lower- and middle-income countries in Latin America including Peru [2, 3]. This increase is attributed to unhealthy diets and physical inactivity [4–6] and current prevention strategies focus on these drivers [4], but early rapid postnatal weight gain is also associated with risk of overweight and obesity [7, 8].

The evidence that early rapid weight gain is a factor in childhood and adult obesity is robust [7–9] but most reported findings are from high-income countries, where child undernutrition is uncommon, or from longitudinal cohorts in low and middle income countries (LMIC) that included children born before the recent increase in obesogenic changes in lifestyle associated with the nutrition transition, and/or concurrent behavioural risk factors were not reported [10].

Breastfeeding has been thought to protect against obesity [11–13] but the one randomized trial of breastfeeding did not show any effect of increased breastfeeding on obesity [14, 15] and another study [16] reported that a longer duration of breastfeeding was not associated with reduced adult adiposity. This last study included populations from LMIC countries with high rates and long duration of breastfeeding. Thus, the possible effect of breastfeeding remains unclear [17].

In addition there are few, if any, longitudinal studies that include rural populations whose lifestyle and growth trajectories are distinct from urban areas but that still experience adult obesity, as in Peru [18].

In Peru, 98.6% of babies are breastfed, with a median duration of 21 months and a median of 4.4 months exclusive breastfeeding [19]. This study presents results from a cohort study of children almost all of whom were breastfed; contrasts rural and urban households; and includes weight, height and waist circumference measurements, as well as concurrent obesogenic risk factors such as physical activity and diet [5].

We hypothesize that rapid weight gain from birth to 1 year (referred to as infant weight gain) and from 1 to 5 years (referred to as early childhood weight gain) would predict overweight and obesity (henceforth referred to as overweight), obesity, BMI for age, and waist circumference at 8 years even when concurrent obesogenic practices are allowed for, but that growth trajectories vary between rural and urban households.

## Methods

Young Lives (YL) is a longitudinal study of children growing up in poverty in four countries [20]. This analysis is confined to 2052 children from a representative sample including all but the 5% richest districts of Peru, due to the study’s focus on poverty. Further details of the sampling strategy have been described [21]. Children aged 6 to 18 months (1 year) were enrolled and household visits conducted between June and December 2001; (Round 2, 2006) when the children were aged 4.5–5.5 years (5 years); and (Round 3, 2009) when the children were aged 7.5–8.5 years (8 years). There was considerable household migration, but families were tracked and 1963 (95.7%) of children were interviewed at 5 years and 1942 (94.6%) at 8 years.

The study collected data on many aspects of the household and the index child; instruments are available online [22]. Relevant to this analysis, information was collected when children were 1 year on breastfeeding (duration and whether the child was currently breastfed) and educational level of parents. Birth weight was taken from the child’s health card (66.7%), or mother’s report. At 1 year children were weighed nude or in light clothing on a calibrated digital balance with 100 g precision (Soehnle: Backnang, Germany) and length was measured using locally-made rigid stadiometers with 1 mm precision by 6 trained supervisors who had passed a standardization exercise. At ages 5 and 8 years all fieldworkers were trained and standardized in anthropometry. Standing height without shoes was measured using locally made instruments accurate to 1 mm and weight was measured in light clothing using digital platform balances as above. No deduction was made for clothing. At 8 years waist circumference was measured to the nearest 1 mm using non-extensible tape placed against the skin midway between the lower rib and superior iliac crest with the tape horizontal to the ground [23]. Data were collected on migration and household location, socioeconomic indicators, the child’s diet including consumption of high calorie purchased food (fast food), hours of sleep and daily activities, and maternal anthropometry.

Information was checked for completion and inconsistencies in the field, and data were entered using range and consistency checks. YL data for rounds 1–3 can be accessed on request online [22]. This analysis is based on data from 1521 children with complete data on birth weight, anthropometry and selected co-variables at all three survey rounds. Of the 1942 children measured in all 3 rounds, 421 were excluded: 224 without birth weight; 14 with a disability that interfered with growth measurement; 47 children were not measured either in round 2 or round 3; 106 mothers were pregnant; and 30 had missing data on covariables.

### Definitions

The dependent variables at age 8 were Z-score of BMI for age, categorized as overweight and obese (>1SD above the median BMI for age) and obese (>2 SD above the median BMI for age), according to WHO reference [24]; and waist circumference.

The independent exposure variables were weight gain over time in relation to the expected weight gain based on the WHO reference standard [24]. Weight gain was measured over two periods: from birth to R1 when children were aged 6–17.9 months (1 year), infant weight gain; and from R1 to R2 when children were 4.5–5.5 year (5 years), early childhood child weight gain. “Rapid weight gain” as a dichotomous variable has been categorized as a change in weight for age of > +0.67 SD. This 0.67 SD represents an increase in weight that on a standard infant and childhood growth chart would be equal to upward crossing of centile bands (2nd, 10th, 25th, 50th, 75th, 90th and 98th) [7]. Waist circumference was analysed as a continuous variable. International obesity task force (IOTF) standards were not used as they are not available for children <24 months [25].

Covariables included in multivariate models were selected because of biological plausibility and prior evidence that they might be relevant to overweight. Continuous variables were standardized by converting them to Z-scores so that the relative weights of the associations could be compared.

The following were retained in the final model because of a significant (*p* < 0.05) association in at least one of the GEE models: sex of the child; birth weight, only in the period from 1 to 5 years as it was collinear with weight gain from birth to 1 year; education level of the mother defined in exclusive categories based on maternal report of highest category of education reached, and BMI of mother when child was 8 years (mean value when available for both rounds). At age 8, child’s height for age Z score, number of days in the previous week that included active exercise such as playing games, bicycling, running; and frequency of eating “fast food” (e.g. crisps, hamburgers, spit roasted chicken, pizza, etc.) and soda drinks. These last two variables were categorized as frequent (more than once a week), less frequent (once a week or fortnight), and infrequent (less than once per fortnight). A wealth index based on housing characteristics, access to services and ownership of consumer durables was included [26]. Other variables included initially but not retained in any model were mother’s age and child’s report of hours of sleep on a normal school day. No adjustment was made for children’s age in months at R1 (ages ranged from 6 to 18 months) nor for the length of time between data collection rounds.

Children living in urban or rural areas were analysed separately because of different lifestyles and environment that may not be captured in the covariables available and because growth trajectories of urban and rural children in Peru have been shown to be different [18]. Analyses with obesity as the dependent variable were restricted to urban children as only 6 rural children were obese. Urban residence was defined as living in a community with >100 houses or in the district capital [19].

### Statistical analysis

Following an initial exploratory correlation analysis, generalized estimating equations (GEE) were used given the cluster design of the study. Unadjusted bivariate models were run for the dependent variables overweight plus obesity, separately for rural and urban children and obesity for urban children only and the independent dichotomous variable weight gain from birth to 1 year and from 1 to 5 years as described above.

To these unadjusted models, covariables were added one by one and retained in the multivariate models when statistically significant (*p* < 0.05), taking care to avoid multiple collinearity. The statistically significant models were compared using the Wald test. The SPSS corrected quasi likelihood under independence model criterion (QICC) was used to determine the most parsimonious models that best explained the relationship between weight change in infancy and the dependent variables. All analyses were done using SPSS version 20 [27]. GEE multivariate analysis of BMI for age against the categorical variable rapid weight gain in the two periods adjusting for the confounders was conducted. GEE analysis was also done with waist circumference as a continuous dependent variable as this is height independent.

## Results

### Rapid weight gain and overweight and obesity at 8 years

Of the 1521 children included in the analyses, 1119 lived in urban areas and 402 in rural areas. Table 1 describes the characteristics of the children according to nutritional status (Additional file 1: Table S1 lists the variables included in the final analysis). At 8 years, 372 urban children were overweight (33.2%) and 127 (11.3%) of these were obese. Overweight and especially obesity were more common in boys. Among rural children 55 (13.7%) were overweight, with only six obese children. Almost all (98.4%) children had been breastfed and 86.6% of children were still breastfeeding at R1. Initial univariate analyses of correlations demonstrated that weight gain in all intervals between birth and 5 years was correlated with BMI Z score at 8 years but with a stronger correlation in urban children (birth-5 years: Pearson correlation *r* = 0.44) compared with rural children (birth-5 years *r* = 0.15) in keeping with different growth trajectories. BMI Z score at 8 years was correlated with waist circumference for urban children (*r* = 0.77) and for rural children (*r* = 0.53).

**Table 1:** Characteristics of the children according to nutritional status

		Normal^a^	Overweight	Obese	Total
		N (%)	N (%)	N (%)	N (%)
Total		1094 (71.9)	294 (19.3)	133 (8.7)	1521
Sex	Female	563 (51.5)	133 (45.2)	49 (36.8)	745 (49.0)
	Male	531 (48.5)	161 (54.8)	84 (63.2)	776 (51.0)
Residence area (aged 1 year)	Urban	670 (61.2)	228 (77.6)	124 (93.2)	1022 (67.2)
	Rural	424 (38.8)	66 (22.4)	9 (6.7)	499 (32.8)
Residence area (aged 8 years)	Urban	747 (68.3)	245 (83.3)	127 (95.5)	1119 (73.6)
	Rural	347 (31.7)	49 (16.7)	6 (4.5)	402 (35.9)
Birth weight	<2.5 kg	67 (6.1)	14 (4.8)	6 (4.5)	87 (5.7)
	≥4.0 kg	58 (5.3)	18 (6.1)	9 (6.8)	85 (5.6)
Breastfed	Breastfed at any time	1079 (98.6)	289 (98.3)	130 (97.7)	1498 (98.5)
Stunting (aged 8 years)	> − 2SD HAZ (height for age Z-score)	241 (22.0)	38 (12.9)	6 (4.5)	285 (18.7)
Urban (% stunted aged 8 years)		117 (48.6)	13 (34.2)	6 (100.0)	136 (47.7)
Rural (% stunted aged 8 years)		124 (51.5)	25 (65.8)	0	149 (52.3)
Stunted (% urban aged 8 years)		117 (15.7)	13 (5.3)	6 (4.7)	136 (12.2)
Stunted (% rural aged 8 years)		124 (35.7)	25 (37.9)	0	149 (37.1)
Soda drinks (aged 8 years)	Frequency ≥1/week	757 (69.2)	226 (76.9)	106 (79.7)	1089 (71.6)
Fast food (aged 8 years)	Frequency ≥1/week	677 (61.9)	220 (74.8)	102 (76.7)	999 (65.7)
		Mean ± SD	Mean ± SD	Mean ± SD	Mean ± SD
BMI for age (BAZ) aged 8 years	8 years	0.04 ± 0.7	1.4 ± 0.3	2.69 ± 0.5	0.54 ± 1.07
Waist circumference aged 8 years	cms	58.47 ± 3.51	64.07 ± 4.55	73.30 ± 6.51	60.84 ± 6.01^b^
Height for age (aged 8 years)		−1.26 ± 0.9	−0.87 ± 1.04	−0.04 ± 1.03	−1.08 ± 1.0
Weight gain in Infancy (1 year-Birth)	Difference in W/A Z-score	−0.07 ± 1.3	0.46 ± 1.3	0.69 ± 1.2	0.1 ± 1.31
Weight gain in childhood (5–1 year)	Difference in W/A Z-score	−0.44 ± 0.85	−0.28 ± 0.88	0.36 ± 1.1	−0.33 ± 0.9
Weight gain in childhood (8–5 years)	Difference in W/A Z-score	0.06 ± 0.4	0.48 ± 0.5	0.86 ± 0.8	0.21 ± 0.5
Mothers education	Years of education	7.5 ± 4.4	9.2 ± 4.5	11.1 ± 3.4	8.15 ± 4.5
BMI of mother (child aged 8 years)		26.44 ± 4.1	28.40 ± 4.4	29.28 ± 4.8	27.07 ± 4.3
Wealth Index^c^ (aged 8 years)		0.53 ± 0.2	0.63 ± 0.2	0.70 ± 0.1	0.56 ± 0.2
Household size (aged 8 years)	No people living in the house	5.45 ± 1.9	5.16 ± 1.9	5.04 ± 1.9	5.36 ± 1.9
Hours asleep (aged 8 years)	No hours normal school day	9.6 ± 0.9	9.58 ± 0.9	9.36 ± 0.8	9.61 ± 0.9
Active exercise^d^ (aged 8 years)	No days in previous 7 days	3.73 ± 2.7	3.63 ± 2.7	2.77 ± 2.4	3.63 ± 2.7

^a^Not overweight or obese

^b^Waist circumference measurements are missing from five children

^c^ Score consisting of housing quality and utilities and ownership of goods

^d^Question asked: “During the last 7 days, on how many days was [Name] physically active for at least 60 min at one time? (Examples for physical activity would be running, biking, dancing, football, digging, carrying water, or other activities)”

The GEE models for rapid weight gain and overweight plus obesity are shown in Tables 2 and 3 for the different age periods, and urban and rural children separately. Infants with rapid weight gain from birth to 1 year were twice as likely to be overweight or obese at 8 years in both urban (adjusted OR 2.06–2.10) and rural (adjusted OR 2.10–2.60) settings (Table 2) and there was a slightly greater risk when the rapid weight gain was from 1 to 5 years (adjusted OR 2.40) for urban children and slightly lower risk at this age for rural children (OR 1.83–2.01) (Table 3). For the more extreme case of obesity, analysed only for urban children, the risk was greater for rapid weight gain from 1 to 5 years (adjusted OR 4.7 to 5.0 (*p* < 0.000) vs. OR 1.35–1.42 from birth to 1 year (*p* value 0.06–0.03) (Additional file 1: Tables S2 and S3).

**Table 2:** Associations between overweight and obesity category in urban and rural children at age 8 years and rapid weight gain from birth to 1 year

	Urban				Rural			
	Null	Unadjusted	Adjusted^a^		Null	Unadjusted	Adjusted^a^	
			Model 1	Model 2			Model 1	Model 2
QICC^b^	1425.13	1388.17	1236.54	1221.06	322.91	318.36	307.74	300.37
Intercept	0.498	0.365	0.353	0.439	0.160	0.126	0.102	0.077
Weight gain >0.67 SD from birth to 1 year		2.27 (<0.001)^c^	2.10 (<0.001)	2.06 (<0.001)		2.29 (<0.001)	2.10 (0.003)	2.60 (0.001)
Sex female			0.55 (<0.001)	0.55 (<0.001)			0.62 (0.001)	0.70 (0.004)
BMI of the mother			1.62 (<0.001)	1.55 (<0.001)			1.49 (<0.001)	1.59 (<0.001)
Height for age (child) at 8 years			1.80 (<0.001)	1.73 (<0.001)				
Wealth index at 8 years				1.50 (<0.001)				
Number of days of active exercise at 8 years			0.87 (0.037)					
*Mothers education level category (compared with incomplete primary school):*								
Further education			1.93 (0.016)				0.89 (0.895)	0.63 (0.629)
Completed secondary school			1.23 (0.500)				1.44 (0.460)	1.16 (0.773)
Incomplete secondary school			1.20 (0.519)				0.53 (0.130)	0.47 (0.077)
Completed primary school			0.88 (0.526)				0.41 (0.015)	0.31 (0.002)
*Frequency of having soda drinks at 8 years (compared with <1 per 2 weeks)*								
> 1 per week (frequent)							3.94 (0.001)	
≥ 1 per 2 weeks (less frequent)							1.73 (0.031)	
*Fast food consumption at 8 years (compared with <1 per 2 weeks)*								
> 1 per week (frequent)								5.11 (<0.001)
≥ 1 per 2 weeks (less frequent)								2.81 (<0.001)

^a^Values given in the table indicate that this variable was adjusted for in the model

^b^Quasi likelihood under independence model criterion

^c^Numbers represent odds ratios and (*p*-values)

**Table 3:** Associations between overweight and obesity category in urban and rural children at age 8 years and rapid weight gain from 1 to 5 years

	Urban			Rural			
	Null	Unadjusted	Adjusted^a^	Null	Unadjusted	Adjusted^a^	
			Model 1			Model 1	Model 2
QICC	1425.13	1387.70	1245.26	322.91	321.24	305.24	303.31
Intercept	0.498	0.425	0.377	0.160	0.141	0.081	0.081
Weight gain >0.67 SD from 1 to 5 years		3.20 (<0.001)^b^	2.40 (<0.001)		2.08 (0.001)	1.99 (0.017)	2.01 (0.018)
Sex female			0.64 (0.001)				
BMI of the mother			1.54 (<0.001)			1.65 (<0.001)	1.63 (<0.001)
Height for age (child) (aged 8 years)			1.80 (<0.001)				
Birth weight			1.11 (0.026)			0.96 (0.761)	
*Mothers education level category (compared with incomplete primary school):*							
Further education			1.95 (0.018)			0.86 (0.865)	0.86 (0.869)
Completed secondary school			1.32 (0.403)			1.07 (0.876)	1.04 (0.938)
Incomplete secondary school			1.19 (0.546)			0.46 (0.058)	0.45 (0.062)
Completed primary school			0.85 (0.450)			0.30 (0.007)	0.29 (0.006)
Number of days of active exercise (aged 8 years)							
*Frequency of having soda drinks at 8 years (compared with <1 per 2 weeks)*							
> 1 per week (frequent)							
≥ 1 per 2 weeks (less frequent)							
*Fast food consumption ay 8 years (compared with <1 per 2 weeks)*							
> 1 per week (frequent)						4.34 (<0.001)	4.31 (<0.001)
≥ 1 per 2 weeks (less frequent)						2.94 (<0.001)	2.92 (<0.001)

^a^Values given in the table indicate that this variable was adjusted for in the model shown

^b^Numbers represent odds ratios and (*p*-values)

In the adjusted models (Tables 2 and 3), greater BMI of the mother was uniformly associated with overweight at 8 years in both time periods and in urban and rural children. Further education, greater height of the child at age 8 and higher birth weight were significantly associated with higher risk of overweight but only in urban children. In the adjusted models for the infancy period, eating “fast food” frequently compared to infrequently was associated with 4–5 times the risk of overweight, and frequent consumption of soda drinks, was a significant risk factor for overweight (OR 3.94), but only for rural children. In analyses of urban obesity for both time periods, physical exercise was significantly protective OR 0.66 (*p* < 0.0001) in all models*.*

### Waist circumference

In GEE continuous analysis unadjusted models (Additional file 1: Tables S4 and S5), the relation between rapid weight gain and this second measure of overweight/obesity was significant in both time periods. In the first adjusted model greater weight gain from birth to 1 year was associated with increased waist circumference (ß coefficient 1.41, *p* < 0.001) in urban children even when including BMI and education attainment of the mother, and frequent fast food consumption. This association was reduced (ß 0.83, *p* < 0.001) but remained significant when height of the child aged 8 years, and number of days of active exercise were included in the model (Additional file 1: Table S4). For rural children the association with greater weight gain was reduced (ß 0.61, *p* < 0.001) when adjusted for BMI of the mother and frequency of consumption of fast food and became nonsignificant when maternal education was included in the adjusted model. Additional file 1: Table S5 shows that while rapid weight gain from 1 to 5 years in the adjusted models in urban children remained significant in this period greater weight gain in rural children was no longer statistically significant when consumption of fast food was included.

## Discussion

These analyses show that rapid weight gain in infancy and in early childhood in both urban and rural children of both sexes in this predominately poor, breastfed population in Peru is associated with overweight and obesity at age 8 years even when other determinants of childhood obesity are taken into account. The association is seen with both weight gain in infancy and in early childhood and is evident in urban and rural children although, while in rural children the greatest effect (OR 2.6) was seen in the adjusted model for rapid weight gain in infancy, in urban children the greatest effect (OR 2.4) was for rapid weight gain in early childhood.

The inference is supported by the association with waist circumference, in urban children but the association is weaker in rural children and becomes non-significant in fully adjusted models at both ages of rapid weight gain. In the case of the rural children concurrent behaviours have a larger impact on waist circumference in the adjusted models. This may be the result of different growth trajectories of rural and urban children or may be due to the lack of discrimination in our indicators of concurrent behaviours in urban children, for instance more than 70% of children drank soda drinks and ate “fast food” at least once a week. In both cases greater height is associated with greater risk of overweight and obesity so it is unlikely that the discrepancy between rural and urban is due to overestimation of overweight due to the fact that the calculation of BMI includes height squared in the denominator.

These findings support evidence that the increasing problem of overweight and obesity in childhood is not only mediated through concurrent behavioural determinants of overweight such as physical activity and diet but that factors such as birthweight, the perinatal hormonal milieu, complimentary feeding practices, and the gut microbiota present in early years including infancy and early childhood also play a significant role [28].

These findings are in keeping with other studies using more accurate techniques such as deuterium [29] and ultrasound measurement of visceral and subcutaneous fat compartments [30], that showed an association of rapid weight gain in early childhood (1–4 years) (although not infancy) with fat mass in adolescence.

These findings contribute to the body of evidence, mainly from developed countries, of the association between early rapid weight gain and overweight/obesity in childhood, adolescence and adulthood [8, 11, 31, 32]. In keeping with other studies these analyses showed a sex difference, but in this case the effect of rapid weight gain was not limited to girls and occurred during infancy, as well as early childhood. This study extends the observations to a recently studied LMIC population with high prevalence and long duration of breastfeeding. Although there is no detailed information on total duration of breastfeeding, 85.8% were still breastfed at R1 (aged 6–18 m). Furthermore, this study also includes indicators of concurrent risk factors for overweight including diet, sleeping patterns and physical activity that were not available for other analyses of rapid weight gain in LMICs [10]. It has also been possible to contrast rural and urban children and show similar results in relation to overweight and obesity measured by BMI, although there are differences with respect to waist circumference. The longitudinal nature of this study will provide further data on longer-term weight trajectories.

There are some weaknesses with our study. Since Young Lives is primarily a study of the effects of poverty it was necessary to include the geographical and social diversity of Peru. A birth cohort was not feasible for this design because of cost and logistics, and thus children were enrolled aged 6–17.9 months and birth weight was based on reported data. Inaccuracy in the birth weights might have weakened the associations and may have added uncertainty to the importance of intrauterine growth restriction (IUGR) in our population. The data on hours of sleep, consumption of soda drinks, fast food consumption and physical exercise is based on self-report using a limited number of questions rather than direct observation or detailed dietary recall. This limits our ability to attribute the observed weight gain to dietary or other factors.

The findings are important as the 8 year old children in our study had a prevalence of overweight 28.1% and 8.6% obesity, consistent with combined prevalence of overweight and obesity in school-age children from Brazil (33.5%) and Mexico (29.3%) [2]. This shows that Peru, along with other countries in South America is in the midst of an increasing problem of overweight and obesity, despite also having a burden of under nutrition, with stunting affecting 32.3% rural and 10.3% urban children under 5 years [19]. Evidence showing that early rapid weight gain in infancy and early childhood can lead to later childhood overweight and obesity, even when almost all babies are breastfed, means that programs and policies need to evolve to ensure that improvements in the quality of the diet improve linear growth without adding additional calories that may induce excessive weight gain.

## Conclusion

In keeping with other studies, we have shown that in a breastfed population from Peru rapid weight gain in infancy and in early childhood in both urban and rural children is associated with overweight and obesity at age 8 years.

## Additional file

Additional file 1: Table S1. Characteristics of the children by Urban and Rural residence. **Table S2.** Associations between obesity in urban children at age 8 years and rapid weight gain from birth to 1 year. **Table S3.** Associations between obesity in urban children at age 8 years and rapid weight gain from 1 to 5 years. **Table S4.** Associations between waist circumference (cm) of urban and rural children at age 8 years and rapid weight gain from birth to 1 year. **Table S5.** Associations between waist circumference (cm) of urban and rural children at age 8 years and rapid weight gain from 1 to 5 years. (DOCX 38 kb)
