# Supplementary material for: Early rapid weight gain and subsequent overweight and obesity in middle childhood in Peru
Source: BMC Obes. 2016 Dec 13;3:55. doi: 10.1186/s40608-016-0135-z (PMC5155392; doi:10.1186/s40608-016-0135-z)
Supplement: Additional file 1: Table S1. — Characteristics of the children by Urban and Rural residence. Table S2. Associations between obesity in urban children at age 8 years and rapid weight gain from birth to 1 year. Table S3. Associations between obesity in urban children at age 8 years and rapid weight gain from 1 to 5 years. Table S4. Associations between waist circumference (cm) of urban and rural children at age 8 years and rapid weight gain from birth to 1 year. Table S5. Associations between waist circumference (cm) of urban and rural children at age 8 years and rapid weight gain from 1 to 5 years. (DOCX 38 kb) [file 40608_2016_135_MOESM1_ESM.docx]

**Table S1: Characteristics of the children by Urban and Rural residence**

| Dependent variable | Urban children  N (%) | Rural Children  N (%) |
| --- | --- | --- |
| Total | 1119 | 402 |
| Overweight and obesity N (%) | 372 (33.2) | 55 (13.7) |
| Obesity N (%) | 127 (11.3) | 6 (1.5) |
| Independent variables (Categorical) N (%) | | |
| Sex – Female | 557 (49.8) | 188 (46.8) |
| Mother’s education level (less than complete primary) | 174 (15.5) | 240 (59.7) |
| Soda drink frequency  One or more times/week N (%) | 852 (76.14) | 237 (58.9) |
| “Fast food” frequency  One or more times/week N (%) | 799 (71.4) | 200 (49.7) |
| Independent variables (Continuous) mean ± range | | |
| BMI-Mother | 27.55 (16.38,47.98) | 25.72 (16.50,39.67) |
| exercise (child) | 3.53 (0, 7) | 3.91 (0,7) |
| height for age (aged 8y) | -0.87 (-3.94,3.33) | -1.67 (-4.29,2.17) |
| hours of sleep | 9.53 (7,13) | 9.75 (6,12) |
| score wealth index | 0.63 (0.06,0.93) | 0.36(0.03,0.84) |
| Birth weight | 3.23 (1,5.2) | 3.11 (1,4.8) |
| Independent variables (Continuous) converted to Z scores mean ± range | | |
| BMI Z score of Mother | 0 (-2.52,4.61) | 0 (-2.44,3.69) |
| Z score for exercise (child) | 0 (-1.32, 1.30) | 0 (-1.44,1.14) |
| Z score height for age (aged 8y) | 0 (-3.14,4,29) | 0 (-2.80,4.10) |
| Z score hours of sleep | 0 (-2.81,3.80) | 0 (-4.25,2.54) |
| Z score wealth index | 0 (-3,42, 1.73) | 0 (-2.26,3.31) |
| Z score Birth weight | 0 (-4.42,3.89) | 0 (-4.13,3.30) |

**Table S2: Associations between obesity in urban children at age 8 years and rapid weight gain from birth to 1 year**

|  | Urban | | | | |
| --- | --- | --- | --- | --- | --- |
|  | Null | Unadjusted | | Adjusted^1^ | |
|  |  |  | Model 1 | | Model 2 |
| QICC | 797.49 | 794.36 | 643.77 | | 643.29 |
| Intercept | 0.106 | 0.098 | 0.043 | | 0.096 |
| Weight gain >0.67 SD from birth to 1y (standardized) |  | 1.910.002) | 1.35(0.075) | | 1.42(0.041) |
| Sex Female |  |  | 0.40(<0.001)^2^ | | 0.40(0.001) |
| BMI of the mother |  |  | 1.55(<0.001) | | 1.51(<0.001) |
| Height for age (child) (aged 8y) |  |  | 2.74(<0.001) | | 2.93(<0.001) |
| *Mothers education level category (compared with incomplete primary school):* | | | | | |
| Further education |  |  | 3.18(0.001) | |  |
| Completed secondary school |  |  | 2.41(0.020) | |  |
| Incomplete secondary school |  |  | 1.96(0.167) | |  |
| Completed primary school |  |  | 2.17(0.076) | |  |
| Number of days of active exercise (aged 8y) |  |  | 0.66(<0.001) | | 0.65(<0.001) |
| *Fast food consumption aged 8y (compared with <1 per 2 weeks)* | | | | | |
| >1 per week (frequent) |  |  |  | |  |
| ≥1 per 2 weeks (less frequent) |  |  | 0.50(0.304) | |  |

^1^ Values given in the table indicate that this variable was adjusted for in the model.

^2^ Numbers represent odds ratios and (p-values).

**Table S3: Associations between obesity in urban children at age 8 years and rapid weight gain from 1 to 5 years**

|  | Urban | | | | | |
| --- | --- | --- | --- | --- | --- | --- |
|  | Null | Unadjusted | | | Adjusted^1^ | |
|  |  | |  | Model 1 | | Model 2 |
| QICC | 797.49 | | 719.79 | 607.15 | | 604.69 |
| Intercept | 0.106 | | 0.084 | 0.078 | | 0.032 |
| Weight gain >0.67 SD from 1y to 5y (standardized) |  | | 7.07(<0.001)^2^ | 4.68(<0.001) | | 5.04(<0.001) |
| Sex Female |  | |  | 0.50(0.006) | | 0.53(0.008) |
| BMI of the mother |  | |  | 1.47(<0.001) | | 1.48(<0.001) |
| Height for age (child) aged 8y |  | |  | 2.75(<0.001) | | 2.43(<0.001) |
| Birth weight (continuous) |  | |  |  | | 1.28(0.016) |
| *Mothers education level category (compared with incomplete primary school):* | | | | | | |
| Further education |  | |  |  | | 3.09(0.002) |
| Completed secondary school |  | |  |  | | 2.49(0.012) |
| Incomplete secondary school |  | |  |  | | 1.84(0.203) |
| Completed primary school |  | |  |  | | 1.84(0.191) |
| Number of days of active exercise (aged 8y) |  | |  | 0.66(<0.001) | | 0.66(<0.001) |

^1^ Values given in the table indicate that this variable was adjusted for in the model.

^2^ Numbers represent odds ratios and (p-values).

**Table S4: Associations between waist circumference (cm) of urban and rural children at age 8 years and rapid weight gain from birth to 1 year**

|  | Urban | | | | | Rural | | | | | |
| --- | --- | --- | --- | --- | --- | --- | --- | --- | --- | --- | --- |
|  | Null | | Undajusted | Adjusted^1^ | | Null | Unadjusted | | Adjusted^1^ | | |
|  |  |  | | Model 1 | Model 2 |  |  | | Model 1 | Model 2 | |
| QICC | 45396.36 | 42904.57 | | 38533.21 | 31095.78 | 5590.59 | | 5438.51 | 5187.60 | | 4382.72 |
| Intercept | 61.78 | 61.78 | | 59.835 | 61.778 | 58.232 | | 58.236 | 57.76 | | 58.132 |
| Weight gain >0.67 SD from birth to 1 year (standardized) |  | 1.50(<0.001)^2^ | | 1.41(<0.001) | 0.83(<0.001) |  | | 0.63(<0.001) | 0.61(<0.001) | | 0.23(0.075) |
| BMI of the mother |  |  | | 1.61(<0.001) | 1.29(<0.001) |  | |  | 0.62(0.005) | | 0.50(0.012) |
| Height for age (child) (aged 8y) |  |  | |  | 2.84(<0.001) |  | |  |  | | 1.47(<0.001) |
| *Mothers education level category (compared with incomplete primary school):* | | | | | | | | | | | |
| Further education |  |  | | 2.93(<0.001) |  |  | |  |  | | -0.01(0.996) |
| Completed secondary school |  |  | | 1.82(<0.001) |  |  | |  |  | | -0.07(0.894) |
| Incomplete secondary school |  |  | | 0.99(0.066) |  |  | |  |  | | -1.07(0.003) |
| Completed primary school |  |  | | 0.31(0.296) |  |  | |  |  | | -0.74(0.222) |
| Number of days of active exercise (aged 8y) |  |  | |  | -0.57(<0.001) |  | |  |  | |  |
| *Fast food consumption aged 8y (compared with <1 per 2 weeks)* | | | | | | | | | | | |
| >1 per week (frequent) |  |  | | 0.91(0.018) |  |  | |  | 1.20(0.010) | | 0.95(0.017) |
| ≥1 per 2 weeks (less frequent) |  |  | | 0.50(0.304) |  |  | |  | 0.83(0.002) | | 0.64(0.001) |

^1^ Values given in the table indicate that this variable was adjusted for in the model. Gender is not included in these models as it was not significantly associated with waist circumference

^2^ Numbers represent the ß coefficient and (p-values).

**Table S5: Associations between waist circumference (cm) of urban and rural children at age 8 years and rapid weight gain from 1 to 5 years**

|  | | Urban | | | | | | | | Rural | | | | | |  |
| --- | --- | --- | --- | --- | --- | --- | --- | --- | --- | --- | --- | --- | --- | --- | --- | --- |
|  | | Null | | Unadjusted | | Adjusted^1^ | | | | Null | | Unadjusted | | Adjusted^1^ | | |
|  | |  | |  | | Model 1 | | Model 2 | |  | |  | | Model 1 | Model 2 |  |
| QICC | | 45396.36 | | 40570.67 | | 30002.95 | | 29471.83 | | 5590.59 | | 5584.62 | | 5316.74 | 4442.21 |  |
| Intercept | | 61.78 | | 61.778 | | 61.778 | | 61.39 | | 58.232 | | 58.232 | | 57.783 | 58.443 |  |
| Weight gain >0.67 SD from 1 to 5y (standardized) | |  | | 2.08(<0.001)^2^ | | 1.44(<0.001) | | 1.45(<0.001) | |  | | 0.14(0.448) | | 0.22(267) | 0.14(0.282) |  |
| BMI of the mother | |  | |  | | 1.21(<0.001) | | 1.18(<0.001) | |  | |  | | 0.69(0.002) | 0.52(0.004) |  |
| Number of days of active exercise (aged 8y) | |  | |  | |  | | 0.62(<0.001) | |  | |  | |  |  |  |
| Height for age (child)  (aged 8y) | |  | |  | | 2.78(<0.001) | | 2.73(<0.001) | |  | |  | |  | 1.56(<0.001) |  |
| Wealth index (aged 8y) | |  | |  | |  | |  | |  | |  | |  |  |  |
| *Mothers education level category (compared with incomplete primary school):* | | | | | | | | | | | | | | | |  |
| Further education | |  | |  | |  | |  | |  | |  | |  | 0.21(0.837) |  |
| Completed secondary school | |  | |  | |  | |  | |  | |  | |  | 0.13(0.809) |  |
| Incomplete secondary school | |  | |  | |  | |  | |  | |  | |  | -.91(0.005) |  |
| Completed primary school | |  | |  | |  | |  | |  | |  | |  | -.52(0.346) |  |
| *Fast food consumption aged 8y (compared with < 1 per 2 weeks)* | | | | | | | | | | | | | | | |  |
| >1 per week (frequent) |  | |  | |  | | 1.03(0.005) | |  | |  | | 1.04(0.018) | |  |  |
| ≥1 per 2 weeks (less frequent) |  | |  | |  | | 0.24(0.583) | |  | |  | | 0.83(0.001) | |  |  |

^1^ Values given in the table indicate that this variable was adjusted for in the model. Gender is not included in these models as it was not significantly associated with waist circumference

^2^ Numbers represent the ß coefficient and (p-values).
